# Assessing network-based methods in the context of system toxicology

**DOI:** 10.3389/fphar.2023.1225697

**Published:** 2023-07-12

**Authors:** Jordi Valls-Margarit, Janet Piñero, Barbara Füzi, Natacha Cerisier, Olivier Taboureau, Laura I. Furlong

**Affiliations:** ^1^ Medbioinformatics Solutions SL, Barcelona, Spain; ^2^ Department of Pharmaceutical Sciences, Faculty of Life Sciences, University of Vienna, Vienna, Austria; ^3^ Université Paris Cité, CNRS, INSERM U1133, Unité de Biologie Fonctionnelle et Adaptative, Paris, France

**Keywords:** systems toxicology, network biology, network propagation, interactome, drug toxicity, drug adverse reactions, multiscale network model

## Abstract

**Introduction:** Network-based methods are promising approaches in systems toxicology because they can be used to predict the effects of drugs and chemicals on health, to elucidate the mode of action of compounds, and to identify biomarkers of toxicity. Over the years, the network biology community has developed a wide range of methods, and users are faced with the task of choosing the most appropriate method for their own application. Furthermore, the advantages and limitations of each method are difficult to determine without a proper standard and comparative evaluation of their performance. This study aims to evaluate different network-based methods that can be used to gain biological insight into the mechanisms of drug toxicity, using valproic acid (VPA)-induced liver steatosis as a benchmark.

**Methods:** We provide a comprehensive analysis of the results produced by each method and highlight the fact that the experimental design (how the method is applied) is relevant in addition to the method specifications. We also contribute with a systematic methodology to analyse the results of the methods individually and in a comparative manner.

**Results:** Our results show that the evaluated tools differ in their performance against the benchmark and in their ability to provide novel insights into the mechanism of adverse effects of the drug. We also suggest that aggregation of the results provided by different methods provides a more confident set of candidate genes and processes to further the knowledge of the drug’s mechanism of action.

**Discussion:** By providing a detailed and systematic analysis of the results of different network-based tools, we aim to assist users in making informed decisions about the most appropriate method for systems toxicology applications.

## 1 Introduction

Proteins exert their function in the context of a molecular network of interactions with other proteins and biomolecules that changes dynamically over time and space. The premise of network biology is that it is possible to dissect biological function by identifying subnetworks or modules representing the concerted action of its components within large-scale networks (e.g., representing gene co-expression, signalling, or protein interactions) (Barabási et al., 2011; Liu et al., 2020). Identification of such modules has been proposed as a cornerstone for precision medicine, as it enables us to gain an understanding of disease mechanisms and move away from organ-based, non-mechanistic disease classifications (Nogales et al., 2022). Disruption of such modules can lead to disease phenotypes by interfering with the function of its members or their relationships. Identification of such modules and how they are perturbed by drugs can also help in the elucidation of the mechanism of drug adverse events. Furthermore, this approach can be applied to unravel the mechanism by which chemical compounds present in the environment and consumer goods lead to toxicity. Network-based approaches hold great promise in the area of systems toxicology to unravel the mechanisms of toxicity (Taboureau et al., 2020), identify mechanistic biomarkers (Callegaro et al., 2023), and assess the effect of genetic variability in the susceptibility of drug adverse reactions (Carss et al., 2022).

Network modules and subnetworks can be identified by different methods including network clustering and community detection, path-finding and network propagation algorithms (Cowen et al., 2017; Liu et al., 2020). However, there is no clear guideline on what the most appropriate method for its application in systems toxicology is to unravel the mechanisms of drug or chemical-induced toxicity. Usually, each methodology is described and evaluated individually in its original publication, in different experimental conditions and using different data and benchmarks, making it extremely difficult to compare results among methods. With the exception of community evaluation initiatives such as the DREAM challenge for the identification of disease modules (Choobdar et al., 2019), and other topics organized under the umbrella of the CAMDA challenge (http://www.camda.info/), there is no community-led, standard evaluation of network-based methods in the context of systems biology or systems toxicology.

In this context, the goal of this study was to perform a systematic evaluation of different network-based approaches that can be applied to uncover the mechanisms of drug-induced toxicity in a systematic manner. To accomplish this goal, we selected a well-characterized drug-induced adverse effect in terms of mechanistic description to be used as a benchmark for comparison of the results obtained (liver steatosis as a result of valproic acid treatment). The interpretation of the results of a network-based approach usually requires analysis and functional interpretation of a large set of genes, which can be facilitated by gene over-representation or enrichment analysis using a variety of functional annotation databases. However, the results of such enrichment analysis can also be cumbersome and tedious due to the large number of statistically significant terms that can be obtained. To overcome this challenge and support the reproducibility of the analysis, we present a systematic approach to analyzing the results of each method, comparing them with each other, and obtaining a consensus among all methodologies. By applying this systematic approach, we analyzed the results obtained by each methodology, as well as a comparison among them. Finally, we combined the results obtained by the different methods to generate a network of genes, biological processes, their interrelations, and associations to the drug and adverse outcome, that can be used to propose novel insights into the biological processes and candidate genes underlying valproic acid (VPA)-induced liver steatosis.

## 2 Materials and methods

### 2.1 Case study: liver steatosis as a result of treatment with VPA

VPA is a short-chain fatty acid, an anticonvulsant that is prescribed to treat epilepsy but also neuropathic pain, migraine, bipolar disorder, spinal muscular atrophy, leukaemia and some solid tumours (Di Pasqua et al., 2022). VPA can cause several adverse effects, such as dizziness, tremor, nausea, endocrinological disorders, obesity, insulin resistance, weight gain, and hepatotoxicity (Farinelli et al., 2015). VPA-hepatotoxicity has three main clinical manifestations: hyperammonemia, acute hepatocellular injury and Reye-like syndrome, all presenting mitochondrial injury and microvesicular steatosis (Di Pasqua et al., 2022). The AOP wiki (AOP Wiki, http://aopkb.org, version March 2022) provides several AOPs describing the different processes that lead to liver steatosis. This knowledge resource was used as a benchmark to compare the results obtained by the network-based approaches applied in our study. Ten AOPs were selected: LXR activation leading to hepatic steatosis (AOP:34), Peroxisomal Fatty Acid Beta-Oxidation Inhibition Leading to Steatosis (AOP:36), AhR activation leading to hepatic steatosis (AOP:57), NR1I3 (CAR) suppression leading to hepatic steatosis (AOP:58), HNF4alpha suppression leading to hepatic steatosis (AOP:59), NR1I2 (Pregnane X Receptor, PXR) activation leading to hepatic steatosis (AOP:60), NFE2L2/FXR activation leading to hepatic steatosis (AOP:61), AKT2 activation leading to hepatic steatosis (AOP:62), NFE2/Nrf2 repression to steatosis (AOP:232), Glucocorticoid Receptor activation leading to hepatic steatosis (AOP:318). From those AOPs we obtained 33 unique genes that will be used for the gene enrichment analysis (Supplementary Table S1). A manual review of the genes involved in each AOP event (Molecular Initiating Event(s), Key Event(s) or Adverse Outcome(s)) was performed. Part of the gene-event association was extracted from the bibliography (Aguayo-Orozco et al., 2019), and the rest of the associations were extracted either from the Event title (when the gene is explicitly named in the KE) or from the bibliography provided in the AOPWiki database.

### 2.2 Human interactome

The human protein interaction network or interactome was obtained from the Multiscale Interactome repository (Ruiz et al., 2021) (https://github.com/snap-stanford/multiscale-interactome, file 3_protein_to_protein.tsv), which integrates data from seven databases. The interactome includes physical interactions among human proteins supported by experimental evidence. Indirect and genetic interactions between proteins and self-interacting proteins were not included (Ruiz et al., 2021). The MI Score from IntAct (a quantitative estimation of the confidence for a given interaction among proteins) was assigned to the edges of the network, resulting in a weighted, undirected interactome. The protein interactome network comprising a total of 17,660 proteins and 387,626 edges was used as a network scaffold for the different network-based approaches considered in this study.

### 2.3 Selection of VPA targets and steatosis-related genes

The human protein targets of Valproic Acid (VPA) were obtained from ChEMBLv29 (Davies et al., 2015; Mendez et al., 2019), Comparative Toxicogenomics Database (CTD) (06/01/2022) (Davis et al., 2023), Papyrus (Version 2, 01/11/2022) (Béquignon et al., 2023), DrugBank (04/01/2022) (Wishart et al., 2018), and DrugCentral (10/03/2022) (Ursu et al., 2016). For ChEMBLv29, we selected associations with type of target “SINGLE PROTEIN” and discarded all associations with potential errors in their validations (data_validity_column from ChEMBLv29), confidence scores below 7 (the confidence score value reflects both the type of target assigned to particular assay and the confidence that the assigned target is the correct one for that assay), and non-active compound (labels not active, and inactive). The associations without both compound activity labels and pChembl values were filtered out. Finally, we converted the UniProt IDs from ChEMBLv29 to NCBI Gene IDs using https://www.uniprot.org/uploadlists/. From CTD, only direct associations (ex: VPA increases the activity of protein Y) and those associated with proteins were selected. The associations due to changes in gene expression, response to a substance, mutagenesis, abundance, oxidation, chemical synthesis, and stability were discarded. Also, we filtered out all contradictory associations (VPA activates/inhibits the same protein). For Papyrus, all VPA-gene associations labelled as “high-quality” were selected. For DrugCentral, we filtered out all VPA-gene associations without activity value and Inchikey.

All sources were combined using the VPA Inchi key. Then, all duplicated interactions, proteins without NCBI Gene ID, and interactions with VPA targets not included in the human interactome network were discarded. Finally, we obtained 70 VPA human protein targets (Supplementary Table S1).

The genes associated with liver steatosis were obtained from DISGENET plus v17.4 (Piñero et al., 2019), including the following UMLS Metathesaurus concepts: Nonalcoholic Steatohepatitis (C3241937), Steatohepatitis (C2711227), Microvesicular hepatic steatosis (C1850415), Macrovesicular hepatic steatosis (C1837256), Diffuse hepatic steatosis (C1849686), Non-alcoholic Fatty Liver Disease (C0400966), Fatty Liver (C0015695). We obtained a list of 1918 genes associated with at least one of liver steatosis concepts, from which 1772 genes were included in the human interactome network. From now on, we will refer to this set of 1772 genes as liver steatosis-associated genes.

The list of VPA targets and liver steatosis genes is provided in Supplementary Table S1.

### 2.4 Network-based methods

#### 2.4.1 Network clustering and cluster analysis

**FIGURE 1:**
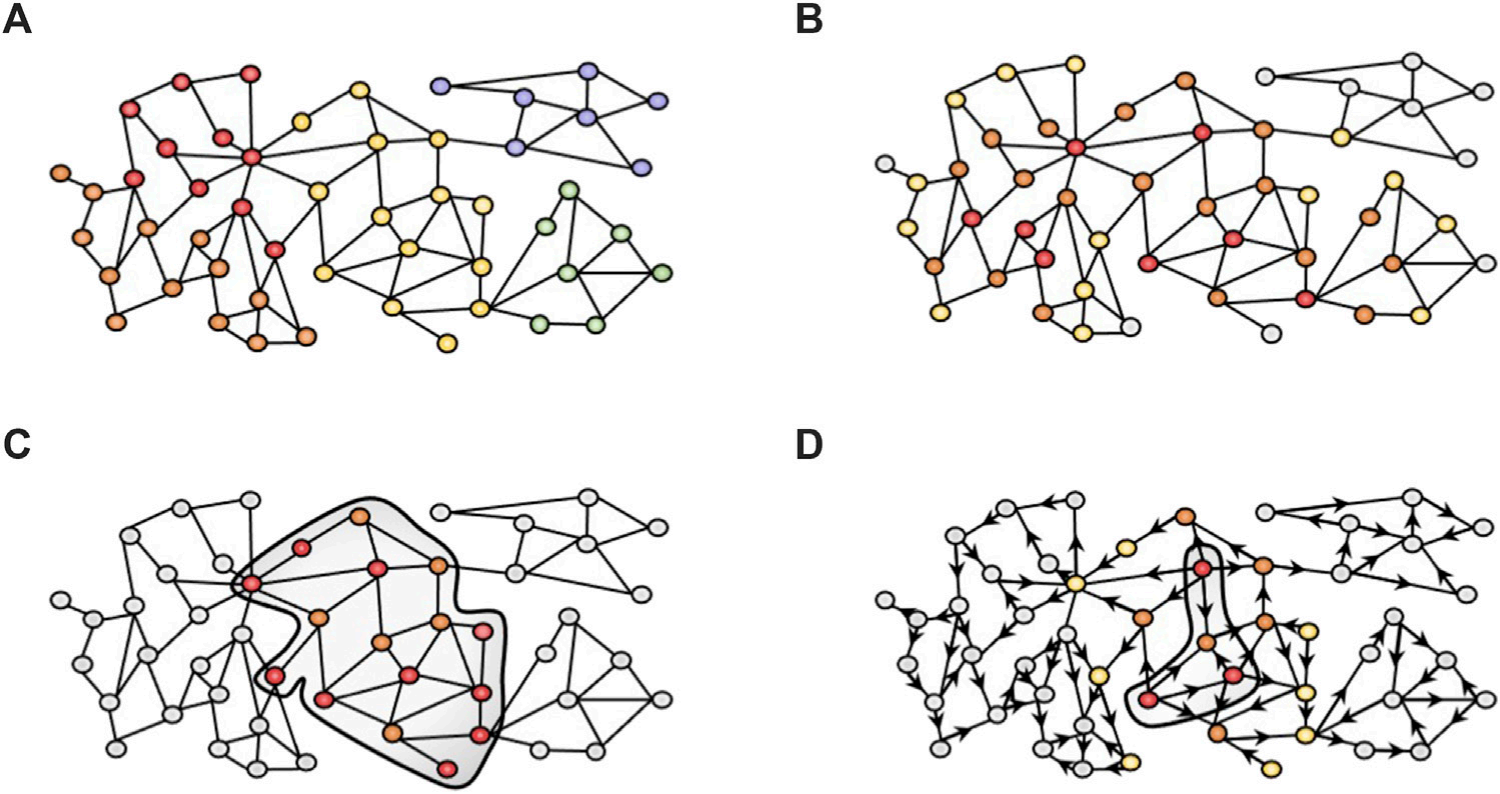
Network-based approaches used in this study. **(A)** Community detection algorithms cluster the nodes in different modules based on the topology of the network. We used the K1 method from MONET. **(B)** GUILD uses a combination of network propagation algorithms to rank candidate genes based on their connectivity to seed genes (red). Genes in close proximity to the seeds are more important (orange) than distant nodes (yellow and grey). **(C)** iPath leverages the Steiner tree algorithm to generate a subnetwork minimizing the linker nodes required (orange) to connect all seed nodes (red). **(D)** Network diffusion profiles provide the visitation frequency of nodes starting from seed nodes (red, for instance, drug targets or disease-associated genes). In the example, orange nodes have higher visitation frequency values than yellow or grey nodes. Then, diffusion profiles are compared to identify those that are more similar and can provide biological insight into the effect of drugs. The Multiscale interactome follows this strategy over a heterogeneous network.

Community detection algorithms are widely used in order to find groups of genes (namely, clusters, modules or communities) that represent coherent biological functions (Figure 1A). Different algorithms can be applied to cluster biological networks. MONET toolbox (Choobdar et al., 2019; Tomasoni et al., 2020) provides the three top-performing clustering methods from the DREAM challenge for disease module identification. Concretely, the K1 method applies a kernel clustering optimization and achieves the best performance to identify disease modules in the DREAM challenge for disease module identification. K1 relies on the Diffusion by State Distance (DSD) method (Cao et al., 2014) that assumes that paths through low-degree nodes are more informative of functional similarity than paths that traverse high-degree nodes (hubs), and therefore overcomes the “ties in proximity” problem of biological networks (Arnau et al., 2005).

The Largest Connected Component (LCC) network was clustered using MONET K1 method using the Intact MI score as edge weight (17,660 nodes and 387,626 edges). The functional annotation of clusters was conducted with g:Profiler (Raudvere et al., 2019) using customized gene sets from DISGENET plus v17.4 (https://www.disgenetplus.com/) and the Comparative Toxicogenomics Database (CTD). Clusters were selected for downstream analysis by their annotation with at least one of the liver steatosis concepts (Diffuse hepatic steatosis, Fatty Liver, Macrovesicular hepatic steatosis, Microvesicular steatosis, Non-alcoholic Fatty Liver Disease, Nonalcoholic Steatohepatitis, and Steatohepatitis), association with Valproic acid (CTD:D014635), and inclusion of VPA targets. Clusters were prioritized using CRANK (version 03/08/2017) (Zitnik et al., 2018), a tool that evaluates the robustness and magnitude of structural features of each module such as the connectivity within and between clusters, and then combines these features into a score. To execute CRANK, we used the MONET output as Community affiliation data (c), the edges without probabilities (MI Scores) as input edged list (i), and the same file with probabilities (ie).

#### 2.4.2 GUILD

GUILD is a network-based prioritization software originally designed for the identification of novel candidate disease genes based on their connectivity to previously known disease genes in the interactome (Guney and Oliva, 2012; Aguirre-Plans et al., 2019). It relies on a protein interactome network and includes a variety of network propagation methods to identify molecular networks underlying human diseases and their comorbidities (Figure 1B). GUILD requires two files, the edge file, which is the interactome file (with MI Scores, see section Human Interactome), and the node file. The node file includes all genes from the edge file, where the seeds are labelled as 1 and the remaining nodes as 0.1. Our experimental setting defined VPA targets and liver steatosis genes as seeds. The rationale behind this design was to identify a network neighbourhood around the set of seed genes (VPA targets and liver steatosis genes) in the interactome network that could represent the protein interaction sub-network that underlies the adverse effect elicited by the drug. Note that other experimental designs could be applied (see below in the iPath section). GUILD was run according to developer recommendations using NetCombo (http://sbi.imim.es/web/index.php/research/software/guildsoftware). All genes with a prioritization value ≥0.8 were selected for the gene enrichment analysis. The seed nodes usually receive the higher scores, followed by other prioritized genes. Note that prioritized genes can be directly or indirectly connected to the seed gene through more than one linker node (Lenselink et al., 2017).

#### 2.4.3 iPath

Among path-finding algorithms, the Steiner tree algorithm (Rintala et al., 2022), generates a subnetwork that minimizes the costs required to connect a given set of seed genes within the network (Figure 1C). The Steiner tree algorithm has been successfully applied to identify the role of protein COS8 in sphingolipid biosynthesis and TOR signalling (Bailly-Bechet et al., 2011) or the association of beta-arrestin 1 and beta-arrestin 2 in the human smooth muscle cells treated by DNase I (Gwinner et al., 2013).

The iPath modelling approach aims at identifying cellular pathways involved in drug toxicity, providing mechanistic hypotheses for drug adverse events (https://bio.tools/ipath_IMIM). iPath implements the Steiner tree algorithm (Rintala et al., 2022), a path-finding algorithm, to obtain a subnetwork from a larger network minimizing the cost required to connect a given set of seed genes within the network. We applied iPath to find the minimum-sized subnetwork that connects the drug targets (used as seeds) through proteins associated with the phenotype of interest (steatosis-associated proteins) and other proteins that are potentially involved in the adverse phenotype (linker proteins). We used all the VPA targets as seeds for iPath, and the genes associated with Steatosis as the linker disease proteins. As the interactome, we used the MSI human interactome file (see below). The subnetwork retrieved by iPath connects all seed proteins and includes as linkers some of the steatosis proteins.

#### 2.4.4 Multiscale interactome (MSI)

Network diffusion approaches, such as random walk, spread a signal through the network emulating a “walker” from one node to another (Cowen et al., 2017; Liu et al., 2020) (Figure 1D). The Multiscale interactome (MSI) (Ruiz et al., 2021) is a good example of the application of biased random walks on a heterogeneous network to model how the effect of a drug or the disease perturbation spread through a hierarchy of biological processes and protein interactions. A diffusion profile is computed by biased random walks that start at the drug or disease node over the heterogeneous network. As such, a drug diffusion profile identifies key proteins and biological processes involved in each drug’s effect. Then, by comparing drug and disease diffusion profiles, the MSI provides an interpretable basis to identify the proteins and biological processes that explain drug effects on disease. We modified the MSI approach to incorporate new datasets, and edge weights and to apply it to drug toxicity (manuscript in preparation). The heterogeneous network underlying the MSI integrates different node types and their interactions: proteins, a full hierarchy of biological processes, drugs, diseases, and their symptoms and manifestations. From the original MSI network implementation (https://github.com/snap-stanford/multiscale-interactome), the data on compound-target and gene-disease associations were updated. Chemical-protein interactions were obtained from ChEMBLv29 database (18/02/2022) (Davies et al., 2015; Mendez et al., 2019) using the assay and activity tables, which include experimental data from the literature. The tables were combined using the “Assay ID”, and the information of targets and drugs through “Molregno ID”. In addition, we included the drug mechanism table, a manually curated dataset that integrates the putative therapeutic targets. The studies that evaluate the effect of compounds on human proteins were selected. Then, the studies focused on protein complexes, chimeric proteins, protein-nucleic acid complexes, protein families, and those studies which evaluate the drug against a protein-protein interaction were removed. All associations with errors in their validations, confidence scores below 7, potential duplicates, uncertain compound activities (according to the following labels: Inconclusive, not active, inactive, not determined, indeterminate, ineffective, lack of solubility, no compound available, no compound detectable, no data, non-valid test, not assayed, not detected, qualitative measurement, precipitate, too insoluble, unable, approximate value, unspecified, uncertain, insoluble, not evaluated, TDE, not tested) were removed. The drug_mechanism from ChEMBLv29 is a manually curated source with putative therapeutic targets, but has no pChembl value. A median pChembl value (7.92) was obtained from those associations that overlapped between the drug_mechanism table and the assay/activity tables (as explained above). This pChembl value was included in all interactions from the drug_mechanism table without the pChembl value. Then, the drug_mechanism table and assay/activity tables were combined. Finally, the pChembl score was divided by 10 to have it on the same scale as the MI score (IntAct).

The uniprotIDs identifying Chembl targets were converted to NCBI Gene IDs using the https://www.uniprot.org/uploadlists/webpage. The gene symbols were downloaded from https://ftp.ncbi.nih.gov/gene/DATA/GENE_INFO/Mammalia/Homo_sapiens.gene_info.gz and included in our dataset using the NCBI Gene ID. To obtain universal compound IDs, the ChEMBL IDs were converted to Inchi keys using the chembl_29_chemreps.txt file downloaded from ChEMBL (https://ftp.ebi.ac.uk/pub/databases/chembl/ChEMBLdb/latest/). The ChEMBL ids were maintained for those compounds without the Inchi key. After harmonizing the IDs, we filtered out the duplicated interactions. Finally, we selected all chemical-target interactions involving proteins included in the 3_protein_to_protein.tsv file (human interactome file).

The Gene-Disease Association (GDA) data was obtained from DISGENET plus (v17.4) (https://www.disgenetplus.com/). We only considered gene-disease associations whose proteins were included in the protein interaction dataset (human interactome file).

We modified the diffusion profile algorithm by incorporating confidence scores on the edges between nodes. The MSI relies on a biased random walk algorithm to propagate the effect of a drug or a disease through the network, using optimized edge weights (*w*_*t*_). The edge weights encode the relative importance of nodes of different types. ***M*** is the biased transition matrix, and each element ***M***_*ij*_ denotes the probability *p*_*ij*_ that a random walker jumps from node *i* to node *j* rather than to another adjacent node of type *t*. We introduced confidence scores (*u*_*ij*_) of the edges between nodes in *p*_*ij*_: for interactions among proteins, the MI score from IntAct; for chemical-protein associations, the pChembl; and for disease-protein associations, the DISGENET score. In the case of the associations between biological process-biological process and biological process-protein, we assigned a score of 0.4. The ***M***_*ij*_ was computed as follows: Let *n*_*t*_ be the number of adjacent nodes of type *t* and *w*_*t*_ the scalar weight of node type *t*.

$$M_{ij}=p_{i\to j}=\frac{\left(w_{t}\;*\;u_{ij}\right)}{n_{t}}$$

The final heterogeneous network comprises 648,822 nodes and 5,134,150 edges. The nodes are from different types: 17,660 proteins, 590,182 drugs, 31,182 diseases, and 9,798 biological processes.

The MSI was executed based on developer recommendations computing the diffusion profiles for VPA and each Steatosis concept (Nonalcoholic Steatohepatitis (C3241937), Steatohepatitis (C2711227), Microvesicular hepatic steatosis (C1850415), Macrovesicular hepatic steatosis (C1837256), Diffuse hepatic steatosis (C1849686), Non-alcoholic Fatty Liver Disease (C0400966) and Fatty Liver (C0015695)).

### 2.5 Identification of the key proteins and biological processes for VPA-steatosis disorders

The diffusion profiles can provide biological insight on how drugs lead to diseases, by pinpointing the key proteins and Biological Processes (BP) involved in the drug effect. These key proteins and BP can be obtained by computing the Treatment Importance (TI), as the product of the visitation frequency of the corresponding protein (or BP) in the drug and disease diffusion profiles as described in (12). We computed the TI for each Steatosis concept (Nonalcoholic Steatohepatitis (C3241937), Steatohepatitis (C2711227), Microvesicular hepatic steatosis (C1850415), Macrovesicular hepatic steatosis (C1837256), Diffuse hepatic steatosis (C1849686), Non-alcoholic Fatty Liver Disease (C0400966), Fatty Liver (C0015695)) and VPA from their respective diffusion profiles. We selected the top 1,000 proteins with high TI for gene enrichment analysis.

### 2.6 Gene enrichment analysis with GO biological processes

To gain insight into the biological processes in which the candidate genes are involved, the gene sets recovered by the 4 network-based methods were analyzed with TopGO (Alexa et al., 2006), and Revigo (Supek et al., 2011). These tools were combined to ease the interpretation of the results of the enrichment analysis, which usually leads to long lists of significantly enriched GO terms. TopGO performs gene enrichment analysis with GO terms taking into account the topology of the GO graph and therefore enables accounting for similarities and redundancies in GO terms. TopGO was executed using the *elim* method, using GUILD scores for the genes in the interactome and the set of genes obtained for each network-based method (from now “candidate genes”). We customized the annotation dataset using the GMT g:Profiler file (gprofiler_full_hsapiens.ENSG.gmt (date 11/10/2022)). The ENSEMBL IDs were converted to NCBI gene IDs using the https://ftp.ncbi.nih.gov/gene/DATA/GENE_INFO/Mammalia/Homo_sapiens.gene_info.gz file. The genes without NCBI gene IDs were discarded. Finally, the file format of the custom annotation dataset, the gene list of the interactome, and the candidate genes were generated according to developer recommendations (https://bioconductor.org/packages/release/bioc/vignettes/topGO/inst/doc/topGO.pdf). TopGO was executed using the Biological Process (BP) category from the Gene Ontology, discarding GO terms with less than 5 annotated genes. Then, GO terms with a Fisher test *p*-value below 0.01 were selected as input for Revigo. In the case of the clusters obtained with MONET, each one of the six clusters selected were analyzed with TopGO individually.

Revigo enables summarizing the results of the enrichment analysis by clustering the resulting GO terms by semantic similarity and providing a representative GO term for each cluster. The result of Revigo is a set of clusters of GO terms. Each GO cluster is represented by the most significant GO term (“representative GO term”). After running Revigo, GO clusters with at least 3 GO terms were used for downstream analysis.

Finally, the similarity among GO clusters was assessed by computing the Jaccard Index among pairs of clusters. GO clusters with a Jaccard Index higher than 0.4 were considered similar.

The Jaccard Index was computed as follows between the set of GO terms from clusters A (A) and the set of GO terms from cluster B (B):

$${JI}_{AB}=\frac{\left|A\cap B\right|}{\left|A\cup B\right|}$$

Comparison among different network-based approaches.

The comparison of gene sets and biological processes obtained from different network methods was performed in R version 4.2.0 using UpSetR (Conway et al., 2017) to evaluate the overlap between datasets/subnetworks generated with each approach.

We selected the candidate genes identified by at least two network-based approaches that were not directly associated with steatosis or VPA targets for further inspection. We refer to these genes as “novel candidate genes”. We used the Human Protein Atlas (HPA) (Uhlén et al., 2015) (https://www.proteinatlas.org/about/download/rna_tissue_consensus.tsv.zip) to assess if these genes are expressed in the liver by selecting genes with an NX score >0 in liver tissue. Finally, the gene enrichment strategy (section “Gene enrichment analysis with GO Biological Processes”) was applied to annotate the novel candidate genes using the Biological Processes terms from the Gene Ontology.

GO terms with a *p*-value of <0.01 and GO clusters with ≥3 GO terms were used for the analysis. The Jaccard Index was computed (details in section “Gene enrichment analysis with GO Biological Processes”) to compare the GO clusters obtained from Revigo. After computing the Jaccard index, the GO clusters were combined in GO groups. The GO groups selected for downstream analysis have to fulfill at least one of the following conditions: 1- Groups containing GO clusters from the four methods, 2- Groups with GO clusters with a Jaccard Index ≥0.4, and 3- Groups that share the “representative GO term” (obtained by Revigo) with at least two GO clusters.

Finally, the GO clusters that belong to the selected groups were compared with GO clusters from the Steatosis AOP using the Jaccard Index.

The R function “logisticPCA” from logisticPCA library was used to perform a Principal Component Analysis (PCA) for count data. Just the groups with one GO cluster for each network method were included in the analysis. The biological processes from groups were the PCA components.

### 2.7 Network representation of novel candidate genes

A representative network was generated to illustrate how the novel candidate genes and selected biological processes are related to the liver steatosis AOPs genes. The novel candidate genes and selected GO groups were used to develop a heterogeneous network including the 33 genes from the liver steatosis AOPs. For the network representation, only the biological processes that overlapped with the 26 novel candidate genes and the 33 genes obtained from liver steatosis AOPs were selected.

The network was generated using as seeds the novel candidate genes, selected GO groups, and 33 genes from the steatosis AOP and the heterogenous network as a scaffold (as described in the section “Multiscale interactome (MSI)”). The shortest paths between the novel candidate genes and VPA targets and liver steatosis genes were extracted using the function “all_shortest_paths” from networkx version 2.3. From all possible shortest paths among selected nodes, we kept those paths including at least one of the liver steatosis AOPs genes. In addition, we selected randomly one shortest path for the novel candidate genes that did not fulfil the previous condition. The novel biological processes were integrated based on the overlap between the genes recovered in the shortest path selection and the GMT file that include the biological process gene sets (section “Gene enrichment analysis with GO Biological Processes”). Cytoscape version 3.9.1 was used for the network representation.

## 3 Results

This study aims at evaluating different network-based methods that can be applied to provide biological insights into how a drug can lead to a disease phenotype. We expect that analyzing in a systematic manner the results obtained from applying them to a case study will help users in selecting the most appropriate method to support research on the mechanisms of drug toxicity. Different network-based approaches can be used to identify the molecular networks perturbed by drugs and chemicals and how they lead to disease phenotypes (Figure 1). We present a framework to compare the results of the different methods in a systematic manner and to propose candidate genes for downstream analysis and validation. Using as a case study liver steatosis induced by VPA, the following network-based methods were evaluated: 1) a community detection algorithm, that clusters the genes based on their connectivity in the interactome followed by cluster selection rules, 2) GUILD, a network-based prioritization method based on network propagation algorithms, 3) iPath, based on the Steiner tree algorithm, that identifies the subnetwork that connects the largest fraction of seed genes within the interactome, and 4) Multiscale interactome (MSI), which relies on comparison of diffusion profiles over a multiscale network (Figure 1; Table 1).

**TABLE 1:** Network-based method and their input data.

Input	MONET	GUILD	iPath	MSI
**VPA targets**	✓*	✓	✓	✓
**Liver steatosis genes**	✓*	✓	✓**	✓
**Interactome network**	✓	✓	✓	X
**Heterogeneous network**	X	X	X	✓

The scaffold network and the input data for each method are indicated. MONET partitions the interactome into clusters and the clusters are selected by their enrichment in liver steatosis genes and Valproic Acid (VPA) targets. The Multiscale Interactome (MSI) compares the diffusion profiles for VPA and liver steatosis from the multiscale network. iPath identifies a subnetwork that connects the seed genes (in this case VPA targets), prioritizing liver steatosis genes as linkers within the interactome. GUILD prioritizes candidate genes using as seed genes both VPA targets and liver steatosis genes.*Used to select clusters annotated with these lists of genes ** Used as preferred linker genes.

**FIGURE 2:**
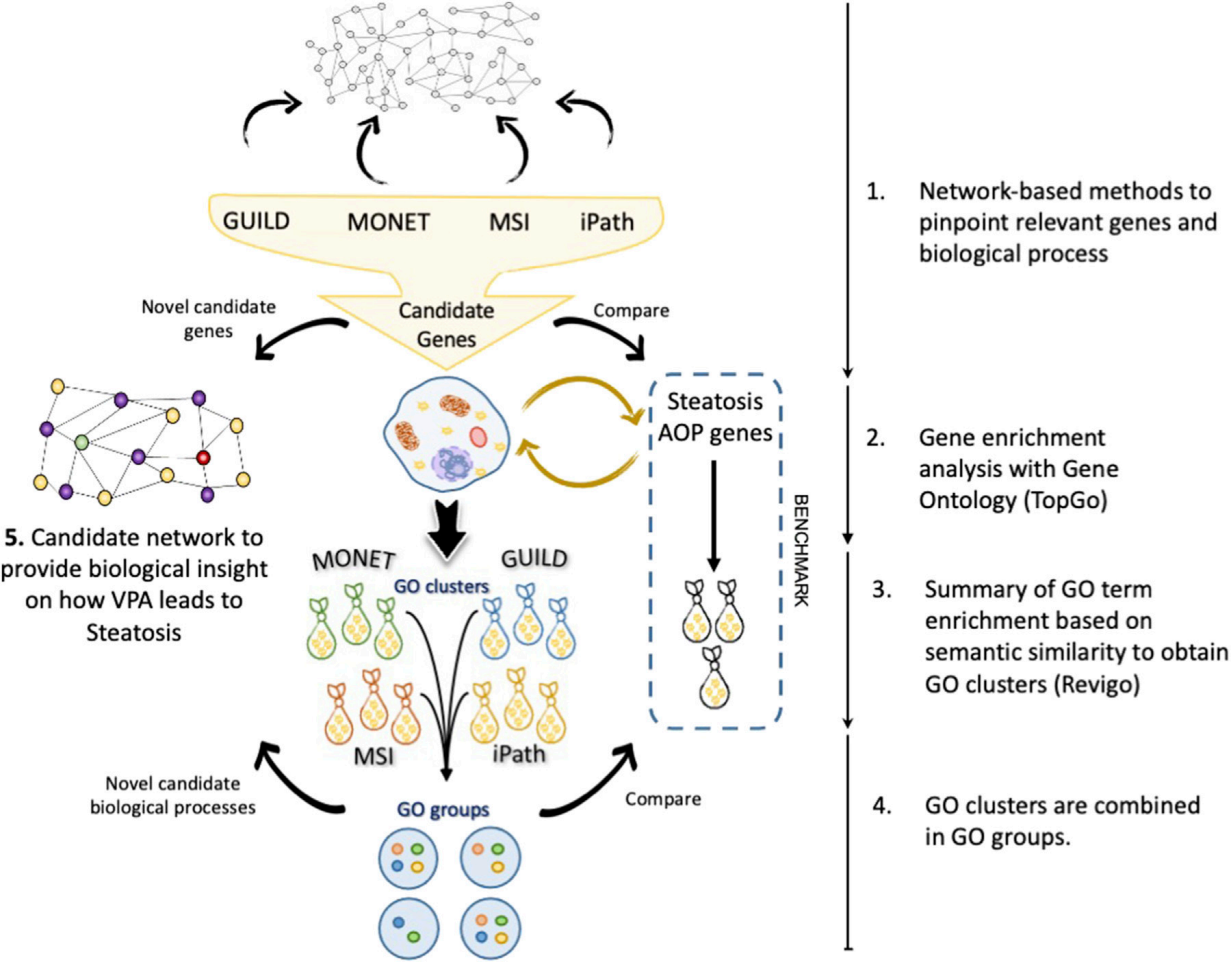
Workflow of the strategy followed in this study. a) The candidate gene sets are recovered after interrogating the network by each tool. A gene enrichment analysis was performed with TopGo, followed by GO term clustering with Revigo. The clusters of GO terms are represented as “bags”. Different GO clusters (bags) can be obtained from each set of genes obtained by each network method. Finally, the GO clusters from different methods are combined in GO groups following the rules exposed in the section “The network approach comparisons”. With novel biological processes (purple) and candidate genes (yellow), a new hypothesis can be created to describe the effect of Valproic acid (VPA) (green) on liver steatosis (red).

Figure 2 presents the proposed workflow to evaluate the results obtained by each network-based method and the strategy followed for the enrichment analysis and comparison with current knowledge on VPA-induced liver steatosis. Using the interactome network as a scaffold, each network-based method provided a set of candidate genes that were analyzed by gene enrichment analysis tools to uncover the biological processes in which these genes are involved. A combination of enrichment tools and filtering processes was implemented to provide a coherent description of the biological processes that represent the lists of candidate genes. In particular, Revigo was used to summarize GO terms into GO clusters based on their semantic similarity. Next, the clusters of GO terms of each network-based method were combined into similar GO groups to obtain a coherent description of GO terms. Finally, a set of novel genes and biological processes are proposed as candidates for the biological mechanism underlying the effect of VPA on liver steatosis (Figure 2).

The rest of the manuscript is organized as follows: first, we present the results of applying each network-based method individually to the case study, second, we present the results of performing a systematic comparison of the results between the different network-based methods, and finally, we propose a candidate network for VPA-induced liver steatosis including genes and biological processes obtained from the consensus of the different methods used in this study. The advantages and limitations of each methodology are presented in the Discussion section.

### 3.1 Clustering approach (MONET)

The clustering of the interactome was performed with the K1 method from MONET, a tool resulting from the DREAM challenge on clustering approaches for disease module identification (Tomasoni et al., 2020). The clustering approach consists of grouping the genes from the network into modules that represent coherent biological functions. The Largest Connected Component of the interactome was used as a network scaffold (17,660 nodes and 387,626 edges). Once a network partition was obtained with the K1 method from MONET, 473 of 1,486 clusters with more than 5 genes were selected for downstream analyses. After the annotation with g:Profiler, we found 26 clusters associated with at least one of the following liver steatosis concepts: Diffuse hepatic steatosis, Fatty Liver, Macrovesicular hepatic steatosis, Microvesicular steatosis, Non-alcoholic Fatty Liver Disease, Nonalcoholic Steatohepatitis, and Steatohepatitis. Finally, 6 clusters were selected based on enrichment with VPA targets (clusters 574, 1,194, 1,237, 1,315, 1,316, 1,330). The robustness of these clusters for downstream analysis was supported by CRANK values, showing high values for all 6 clusters (between 0.87 and 1). Particularly, cluster 1,194 has a CRank of 1, indicating that it is stable against network perturbations. Note that this experimental design assumes that clusters of interest must contain both drug targets and disease genes.

The selected 6 clusters contain 6% of Liver Steatosis genes and 16% of VPA targets (Table 2). After annotating with TopGO the 6 clusters individually, 548 GO terms with a *p*-value <0.01 were obtained (Table 3). Clusters 1,194 and 1,330 have the most similar GO terms with 13 overlapping annotations. In particular, the GO term “positive regulation of transcription by RNA polymerase II (GO:0045944)” is the most significant in both clusters, which agrees with the results obtained with the MSI approach. The other 4 clusters are associated with different terms, for instance, acute-phase response (GO:0006953) for cluster 1,315, protein autophosphorylation (GO:0046777) for cluster 1,237, mitochondrial translation (GO:0032543) for cluster 574, and negative regulation of endopeptidase activity (GO:0010951) for cluster 1,316.

**TABLE 2:** Number of genes recovered by each method and comparison with VPA and steatosis genes.

Network method	Total genes	VPA targets recovered (n = 70)	Steatosis proteins recovered (n = 1772)	Unique VPA and steatosis targets recovered (n = 1808)	Overlap with steatosis AOP genes (n = 33)	New gene candidates for steatosis induced by VPA
MONET	421	11 (16%)	111 (6%)	114 (6%)	10 (30%)	307 (73%)
MSI	1,000	29 (41%)	571 (32%)	576 (32%)	25 (75%)	424 (42%)
iPath	126	69 (99%)	85 (5%)	120 (7%)	5 (15%)	6 (5%)
GUILD	823	25 (36%)	817 (46%)	823 (46%)	24 (72%)	0 (0%)
**Totals (unique)**	1761	69 (99%)	1,015 (57%)	1,050 (58%)	29 (88%)	710 (40%)

The column “Total genes” shows the number of genes recovered by each method, while the columns that follow show the number of genes that overlap with each gene set: VPA targets, liver steatosis genes, and steatosis AOP genes. The last column shows the number of gene candidates obtained by each method, with percentages shown with respect to the total number of genes recovered by each method.

**TABLE 3:** Gene enrichment analysis with the Gene Ontology (Biological Process).

Network method	Num genes	Num biological processes	Num biological processes clustered	Num of GO clusters (Revigo)	Num GO clusters ≥3 GO terms	Num biological processes in GO clusters ≥3 GO terms
MONET	421	548	196	80	23	82
MSI	1,000	1814	1,259	307	172	989
iPath	126	402	182	63	29	114
GUILD	823	1,529	1,017	253	146	803

The statistics of the enrichment analysis results are shown, indicating the number of significant terms obtained for each list of genes. The Multiscale Interactome (MSI) and GUILD methods recover more GO clusters with at least 3 GO terms. Details on the GO terms obtained for each method are provided in Supplementary Table S2 and Supplementary Table S3.

Revigo was used to cluster the GO terms in GO clusters. 196 GO terms out of 548 were grouped in 80 GO clusters, where 23 clusters had ≥3 GO terms (Table 3). After comparing the overlap between 23 GO clusters, the Jaccard Index was 0 for all clusters, showing that the 6 clusters represent distinct biological functions. For example, the largest GO clusters for each of the 6 MONET clusters were the following ones: For cluster 574 the mitochondrial translation (GO:0032543), for cluster 1,194 the positive regulation of fatty acid oxidation (GO:0046321), for cluster 1,237, toll-like receptor 4 signaling pathway (GO:0034142), for cluster 1,315, the regulation of triglyceride catabolic process (GO:0010896) and complement activation, classical pathway (GO:0006958), for cluster 1,316, omega-hydroxylase P450 pathway (GO:0097267), and cluster 1,330 the RNA polymerase II preinitiation complex assembly (GO:0051123).

Finally, the results of the GO enrichment analysis of the MONET clusters were compared with the enrichment analysis of liver steatosis AOPs. 69 of 178 GO terms annotated with TopGO using the liver steatosis AOPs genes overlapped with the results of the enrichment analysis of the 6 MONET clusters. Cluster 1,194 shared 46 of the 69 GO terms, and included the most significant GO terms recovered by the gene enrichment of liver steatosis AOPs. Only one GO cluster has a Jaccard Index above 0.4 (Jaccard Index 0.5, cluster 1,194, associated with fatty acid oxidation and the biosynthetic process of fatty acids and lipids) when compared to the GO clusters from the liver steatosis AOPs genes.

### 3.2 GUILD approach

We applied GUILD using as seeds the list of VPA targets and liver steatosis genes to prioritize genes potentially involved in VPA-induced liver steatosis (Table 1). A set of 823 genes was obtained with a prioritization score ≥0.8. From these 823 genes, 25 genes overlapped with VPA targets and 817 with liver steatosis-associated proteins (Table 2). Compared to other approaches, all genes provided by GUILD were already associated with VPA or liver steatosis. Finally, 24 of 33 genes from the liver steatosis AOP were recovered with the GUILD approach.

By gene enrichment analysis a list of 1,529 biological processes with a *p*-value ≤0.01 was obtained, the three biological processes with high *p*-values were also obtained with the MSI approach (negative/positive regulation of transcription by RNA polymerase II and positive regulation of gene expression (GO:000122, GO:0045994, GO:0010628)). Following the analysis with Revigo, 1,017 of 1,529 biological processes were clustered in 253 modules, with 58% of them having ≥3 GO terms (Table 3). Particularly, the largest cluster sizes with 18 GO terms, are associated with a glycolytic process, lipid biosynthetic process, and ERK1/2 activity.

One-hundred-fifteen out of 178 GO terms overlapped between Steatosis AOP gene enrichment and GUILD. Fourteen out of fifteen GO clusters from Steatosis AOP had some GO term overlapped with 253 GO clusters from GUILD. Concretely, 5 GO clusters with a Jaccard Index above 0.4 were associated with cholesterol homeostasis, response to hormones, insulin secretion, ethanol response, and cholesterol transport.

### 3.3 iPath approach

iPath requires a scaffold network, a set of seed genes, and another set of genes to be prioritized as linkers (Table 1). The chosen experimental design resulted in a subnetwork that captures most VPA targets prioritizing connections through liver steatosis genes. As such, the resulting subnetwork of 126 genes, which contains most VPA targets present in the human interactome, reflects the protein network neighbourhood of the targets of VPA. As a result, a network of 126 genes including most VPA targets (69/70) (note that the missing gene, UGT1A3, was one not included in the scaffold network), and 85 of 1772 Steatosis genes were obtained (Table 2). Six genes of the iPath subnetwork have not been previously associated with Steatosis, or VPA (Table 2). Five of the 33 Steatosis AOP genes were included in the iPath subnetwork (Table 2).

After the gene enrichment analysis, 402 biological processes were obtained with a *p*-value of 0.01 (Table 3). The most significant biological process was the xenobiotic catabolic process (GO:0042178) with a *p*-value of 1.3e-13, which has been associated with fatty liver diseases [47] followed by the estrogen metabolic process (GO:0008210) (*p*-value 1.8e-13). After the analysis with Revigo, 182 GO terms were clustered in 63 modules, where 46% of them had ≥3 GO terms (Table 3). Three clusters had 7 GO terms where the GO representative is associated with the neuronal action potential (GO:0019228), positive regulation of glycolytic process (GO:0045821), and regulation of heart rate by cardiac conduction (GO:0086091).

Comparing the gene enrichment between the Steatosis AOP gene set and iPath, 50 of 178 GO terms annotated with the Steatosis AOP gene set were also annotated with the iPath approach. On the other hand, 7 of 15 GO clusters from Steatosis AOP had some GO term in common. Two of them had a Jaccard Index above 0.4. One cluster is associated with the metabolic process of estrogen, cholesterol, steroid, and androgen (Jaccard Index of 0.75). The second is associated with the response of ethanol and prostaglandin (Jaccard Index of 0.5).

### 3.4 Multiscale interactome approach (MSI)

MSI relies on comparing the diffusion profiles of drugs and diseases that are obtained from the heterogeneous network, which integrates information on drug targets, disease-associated genes, protein interactions, and a hierarchy of biological processes. By comparing diffusion profiles, it is possible to prioritize the genes and biological processes that are relevant to a particular drug effect. By comparing the diffusion profiles of VPA and liver steatosis, the top-1000 genes and biological processes were selected based on their values of the Treatment Importance (TI) (further details in the section “Multiscale interactome (MSI)”). One thousand genes represent 0.15% of each diffusion profile. Smaller sets of genes could be selected for downstream processing, but we decided to select a large number of genes to enable covering a large fraction of seed genes and to emulate situations of analysis of omics datasets where a large number of genes are obtained for further analysis. From this selection, 32% of the top-1000 genes overlapped with liver steatosis genes, while 41% overlapped with VPA targets. Seventy-five percent of Steatosis AOP genes were included in the top-1000 gene set (Table 2). Forty-two percent of the top-1000 genes are not included in the VPA targets, liver steatosis genes, or liver steatosis AOP gene sets, and therefore could be novel candidate genes involved in liver steatosis induced by VPA (Table 2). The set of 1,000 genes was characterized by enrichment analysis to gain insight into the biological processes in which they are involved.

The gene enrichment analysis resulted in 1814 GO BP terms (*p*-value of 0.01), with the most significant terms related to the regulation of gene expression: negative/positive regulation of transcription by RNA polymerase II (*p*-value < 1e-30), positive regulation of gene expression (*p*-value 1.8e-29), positive regulation of miRNA metabolic process (*p*-value 2.1e-28). Most of the GO terms (1,259 of 1814) were clustered in 307 groups by Revigo, where 56% of the clusters have a size ≥3 GO terms (Table 3). The largest cluster includes 25 GO terms and is associated with “positive regulation of proteasomal ubiquitin-dependent protein catabolic process” (GO:0032436), followed by a cluster of 21 GO terms associated with “positive regulation of fatty acid beta-oxidation” (GO:0032000) process. These processes have been reported as associated with liver steatosis (Koo, 2013).

Finally, we contrasted the biological processes obtained from the top-1000 genes with those obtained by gene enrichment of the liver steatosis AOPs genes. Overall, 64% of GO terms enriched from liver steatosis AOPs genes overlapped with the GO terms enriched from the top-1000 genes. From the 15 GO clusters obtained from liver steatosis AOPs, 14 GO clusters were found in common with the ones obtained from the MSI top-1000 genes. Three clusters have a Jaccard index higher than 0.4, where the cluster with the highest Jaccard index (0.75) is associated with fatty acid, triglyceride, and cholesterol homeostasis, followed by a cluster associated with response to Vitamin A and another with ethanol.

## 4 Comparison among methods

### 4.1 Comparison at the gene level

The number of genes recovered by each method is shown in Table 2. MSI and GUILD are the methods that result in a higher agreement with the liver steatosis AOPs, both in terms of genes and GO Biological Process (BP) annotations. According to MSI, not all liver steatosis-associated genes nor the VPA targets are important for VPA-induced liver steatosis (only a fraction of liver steatosis genes and VPA targets are recovered in the top-1000 genes selected by Treatment Importance). We can arrive at a similar conclusion by analyzing the results from GUILD. The ample coverage of both approaches compared to the other ones tested might be explained by the threshold resulting in large lists of genes (1,000 genes for MSI and 823 genes for GUILD). Another factor that could explain the larger coverage of MSI compared with other approaches is its ability to identify genes as important for the drug effect on the disease despite not being in the close network neighbourhood of the seed genes, a fact that has been reported to be important to explain the effect of drugs on diseases (Ruiz et al., 2021). Due to the ability of the Steiner tree algorithm to connect the highest fraction of the genes used as seed, iPath recovers a comparatively smaller network and gene set which could explain the very low coverage of the benchmark dataset. MONET, on the other hand, has the potential of capturing every gene included in the network but the prioritization and selection steps condition the coverage of the benchmark dataset. MONET captures a smaller fraction of VPA targets and steatosis genes compared with the other approaches (with the exception of iPath for steatosis genes). The small fraction of VPA targets recovered might be due to the experimental design in the clustering approach, which started by selecting disease-enriched clusters. This step might result in losing clusters that contain drug targets but not disease genes. The large size of the steatosis gene list might explain the low coverage by all the methods. It is surprising that the clustering approach recovers a small fraction of liver steatosis AOPs genes (30%). By analyzing the results at each selection step carefully, we found that 29/33 of the liver steatosis AOPs genes were distributed in 21 of the 473 clusters with more than 5 genes, indicating that the low coverage is the result of the subsequent filters applied.

Regarding the set of genes suggested as candidate genes by each method, we note that GUILD does not provide any candidate gene, probably due to the threshold used to select the gene set. iPath suggests only 6 candidate genes, coherent with the results of a small subnetwork of 126 genes. MONET and MSI are the methods that provide a larger number of candidate genes (Table 2).

**FIGURE 3:**
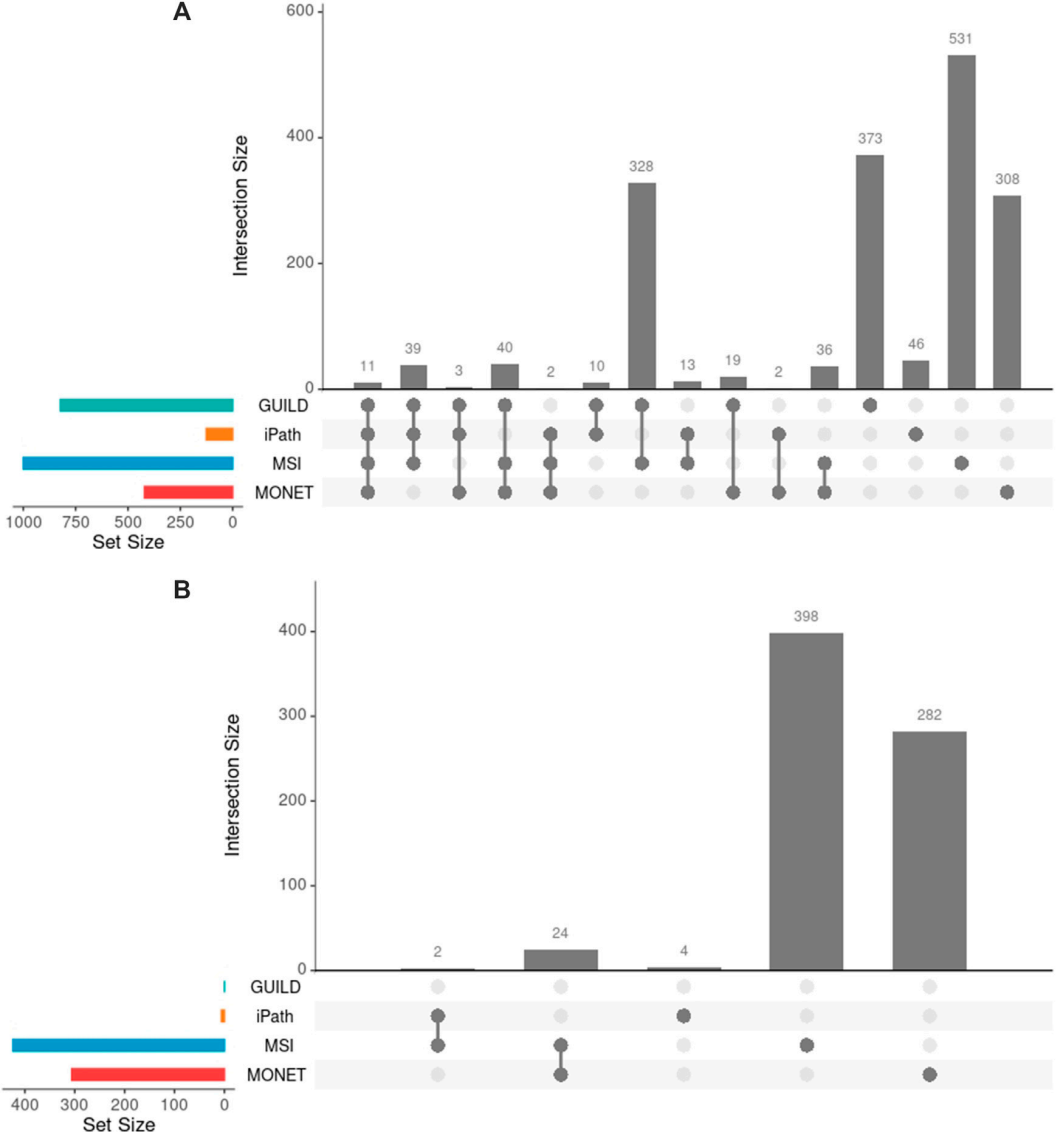
Comparison of genes across methods. **(A)** Genes obtained by each method and their overlap. Few overlaps were appreciated across all methods, 29% of 1761 genes overlapped by at least two methods. **(B)** Novel candidate genes obtained by each method and their overlap. Note that GUILD did not propose any candidate gene.

After interrogating the network using the four methods, 503 of 1761 unique genes were recovered by two or more strategies, and 11 genes were detected by all approaches (Figure 3A). The highest overlap in terms of shared genes was obtained between GUILD and MSI (328 shared genes, Figure 3A). This set of 328 genes was also shared with the liver steatosis-associated genes. Seven of 70 VPA targets were detected by all methods and 16 by three.

GUILD is the tool that recovers most disease-associated genes (818/1772) and iPath recovers the lowest fraction (85/1772). Considering the genes detected by four or three methods respectively, 10 of 11, and 81 of 84 targets overlapped with Steatosis-associated proteins (Figure 3A), showing consistency of gene findings with Steatosis. Most of the liver steatosis AOP targets were detected by at least two approaches (22 of 29), demonstrating that this selection criterion can be used to select candidate genes for downstream analyses.

**FIGURE 4:**
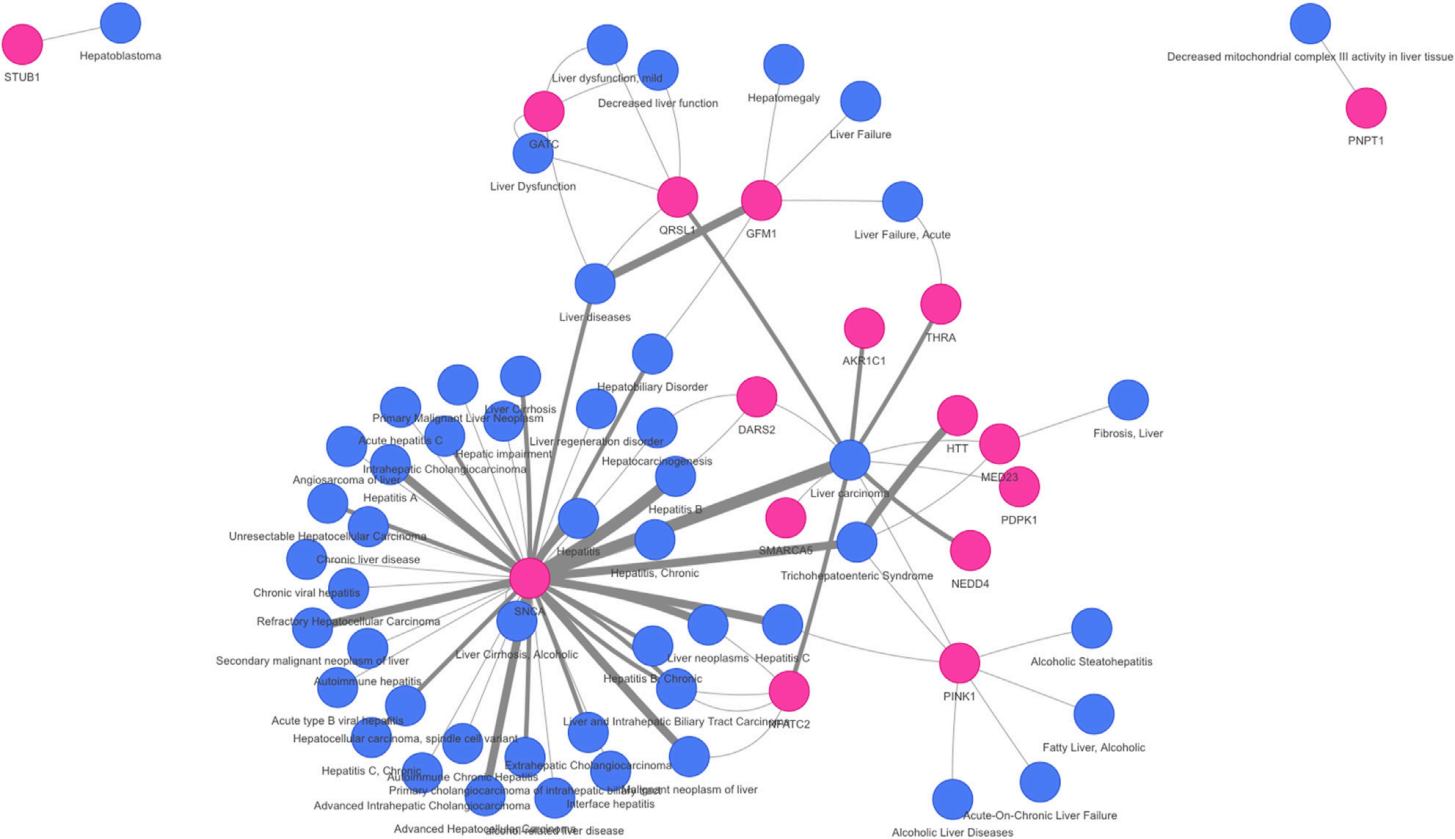
Association of novel candidate genes with liver diseases and phenotypes. A network representation of the gene-disease association of 16 of the candidate genes with liver diseases was obtained using DISGENET plus (https://www.disgenetplus.com/) and the disgenetplus2r package (https://medbio.gitlab.io/disgenetplus2r/). The width of the edges is proportional to the DISGENET plus score.

On the other hand, the network approaches can be used to propose novel candidate genes. In the present study, 710 genes retrieved by at least one of the methodologies have no previous direct association with VPA and/or Liver Steatosis (Table 2). MSI and MONET were the approaches that recover more candidate genes. In addition, 26 of these genes were detected by two network methods: 24 were detected by MSI and MONET, and 2 by MSI and iPath (Figure 3B). The genes THRA and NCOR2 were included in the 1,194 cluster and are also detected by MSI, suggesting that these genes could be relevant for the effect of VPA on liver steatosis. The thyroid hormone is involved in the regulation of lipid and glucose metabolism, and a knock-out mouse of the thyroid receptor alpha is protected from diet-induced hepatic insulin resistance (Jornayvaz et al., 2012). The protein encoded by the NCOR2 gene is part of the HDAC3 complex, which is involved in gene expression regulation in hepatocytes in response to environmental stimuli (Liang et al., 2019). In addition, gene expression analysis shows that the set of 26 genes are expressed in the liver, with 20 of them having a medium level of expression (data not shown). Finally, there is evidence of association with a variety of liver phenotypes for an important fraction of the candidate genes (Figure 4), providing additional evidence for their validity as candidates for the effect of VPA on liver steatosis.

### 4.2 Comparison at the level of biological processes

**FIGURE 5:**
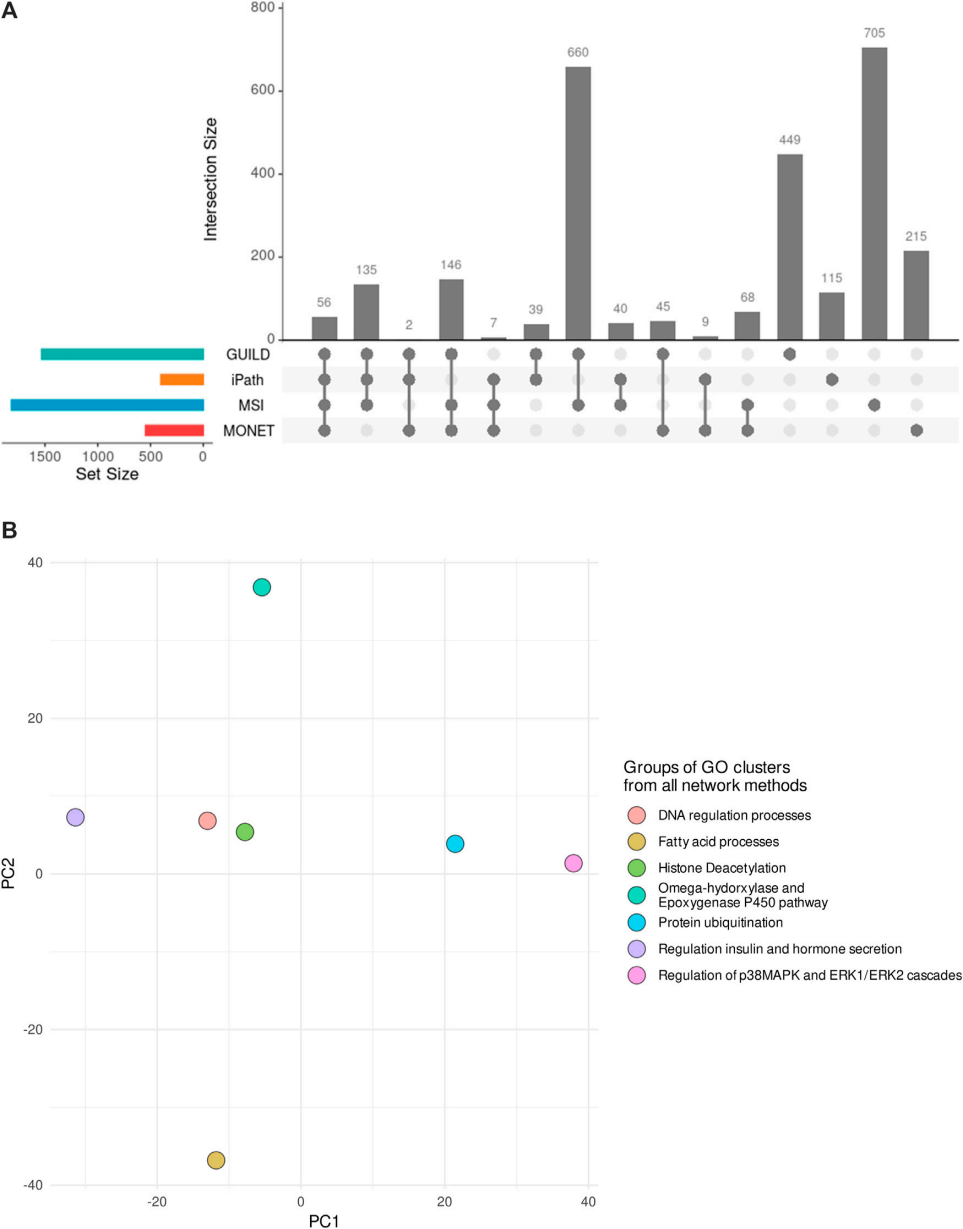
Comparison of Biological Processes obtained by each network method and their combination in GO groups. **(A)** Biological Processes significantly enriched in each gene list of the network-based methods and their comparison. **(B)** PCA depicting the 7 GO groups of GO clusters supported by the four network-based methods. Each dot represents the group of GO clusters from Revigo. The rules followed to generate the GO groups are described in the section “Comparison among different network-based approaches”. The PCA shows three different organizations across the 7 GO groups, suggesting different modes of action for the Steatosis development.

Considering all network methods, a total of 2,691 biological processes with a *p*-value <0.01 were obtained after the gene enrichment analysis, and 1,207 were common at least by two strategies. The largest number of significantly enriched GO terms was obtained for GUILD and MSI (Table 3), probably due to the larger gene sets. The larger gene sets in combination with the 328 genes shared between both approaches (Figure 3A) could explain why GUILD and MSI share 660 biological processes (Figure 5A). Furthermore, 56 biological processes were shared across all methods (Figure 5A).

Contrasting the annotation with Steatosis AOP gene enrichment, 118 of 178 liver steatosis AOPs GO terms overlapped at least with two strategies. In addition, 60 of 118 Steatosis GO terms were detected at least by three methods, suggesting that the same biological process obtained from different network methods can be considered for downstream analysis.

Revigo was used to organize and summarize the results of the enrichment analysis and find a representative subset of biological processes. 1,400 of 2691 GO terms were clustered in 307 GO modules with a size of ≥3 GO terms. Comparing the clusters obtained by each network method, 220 of 307 GO modules have some biological processes in common.

The GO clusters were combined in GO groups based on one of the following three conditions: GO Groups containing GO clusters from the four methods, Groups with GO clusters with a Jaccard Index ≥0.4, and Groups that share the “representative GO term” with at least two GO clusters. As a result, 149 GO clusters were considered similar and grouped in 92 GO groups. Note that 7 groups included GO clusters from the four network-based methods. The 7 GO groups were formed by 25 GO clusters which included 135 GO terms (a list of terms is provided in Supplementary Table S2 and Supplementary Table S3). The 7 GO groups are associated with 1) Omega-hydorxylase/Epoxygenase P450 pathway, 2) Regulation of insulin and hormone secretion, 3) Regulation of p38MAPK and ERK1/ERK2 cascades 4) Histone Deacetylation, 5) Fatty acid processes, 6) Protein ubiquitination, and 7) DNA regulation processes (Figure 5B). The seven groups can be distributed in three regions in the PCA (Figure 5B), based on the similarity among the biological processes they represent. The closest groups are the ones representing DNA regulation process and Histone Deacetylation.

From the 15 liver steatosis AOPs GO clusters, 14 have GO terms in common with the 25 GO clusters from all the network methods. In addition, the GO groups associated with positive regulation of fatty acid beta-oxidation, positive regulation of insulin secretion, and fatty acid beta-oxidation included five GO clusters from the liver steatosis AOPs (representative GO terms: fatty acid beta-oxidation (GO:0006635), positive regulation of fatty acid beta-oxidation (GO:0032000), regulation of insulin secretion (GO:0032024), negative regulation of fatty acid metabolic process (GO:0045922), and positive regulation of hormone secretion (GO:0046887). These similarities suggested that the combination of results from different methods could help in the identification of biological processes underlying the effect of VPA on liver steatosis.

### 4.3 Network representation of novel candidate genes and biological processes for VPA-induced steatosis

We combined the results of the different approaches to propose novel candidate genes and biological functions that can provide biological insights into the mechanism by which VPA leads to liver steatosis. A representative network was generated to show how the novel candidate genes and selected biological processes are related to the liver steatosis AOPs genes, VPA targets and liver steatosis genes.

The network was generated using as seeds the 26 novel candidate genes, 25 GO clusters from the 7 GO groups (representing 135 GO terms), and 33 genes from the steatosis AOP. Using the heterogeneous network as a scaffold, the VPA targets and liver steatosis were connected using shortest paths (for more details see Methods). Fourteen of the 135 biological processes included genes that overlapped with at least one of the 26 novel candidate genes and 33 genes from the liver steatosis AOPs. These 14 biological processes are mainly associated with protein regulation, kinase activity, histone deacetylation, and phosphorylation processes.

**FIGURE 6:**
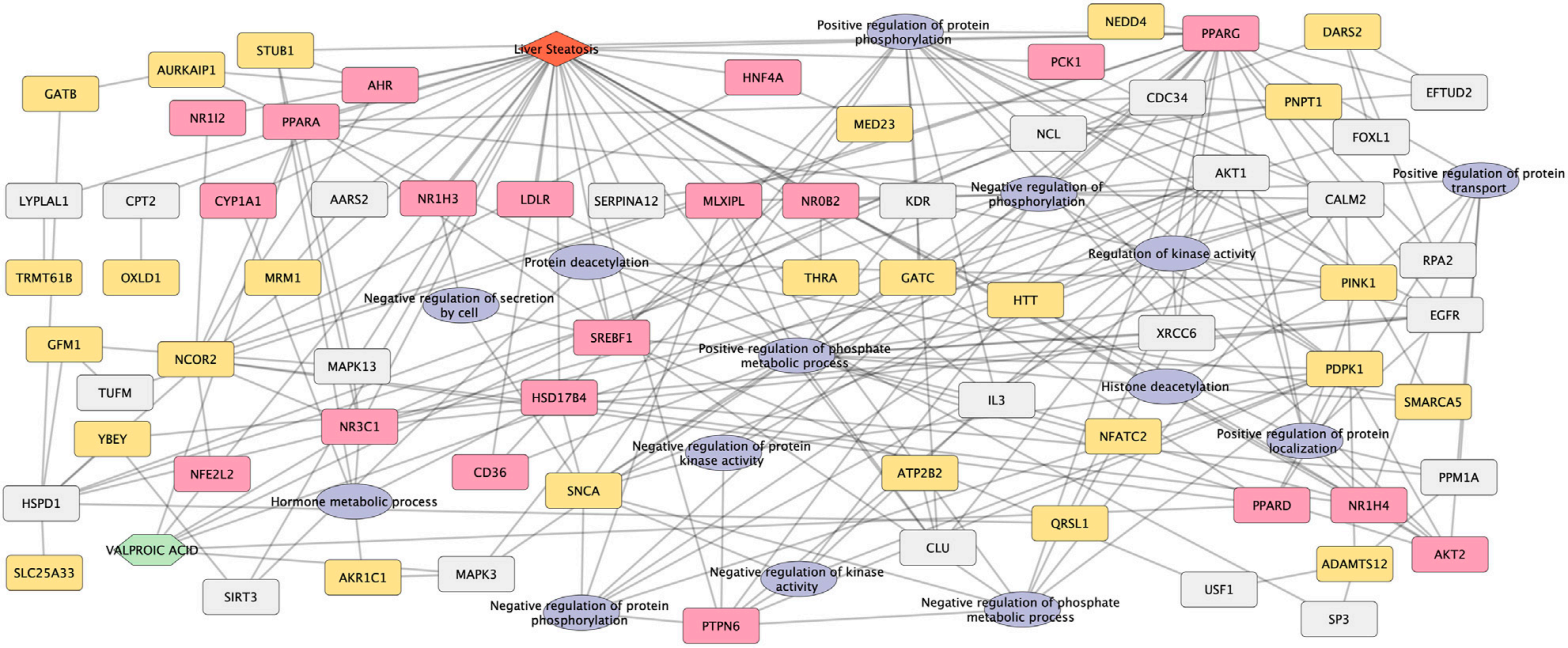
Network representation of novel candidate genes and biological processes for Valproic acid-induced liver steatosis. The network integrates the novel candidate genes and biological processes obtained from the combination of the four network methods. Both 26 novel candidate genes and 14 biological processes are associated with the liver steatosis AOPs genes (20 genes), VPA targets and liver steatosis genes through linker genes (23 genes, in grey). The network contains 86 nodes and 218 edges.

A network was created from the 26 novel candidate genes, the 14 biological processes connected by 23 linker genes (Figure 6).

## 5 Discussion

This study aims at evaluating different network-based methods that can be applied to provide biological insights into how drugs lead to diseases. The approaches evaluated in this study are based on different network medicine assumptions (Barabási et al., 2011; Liu et al., 2020) and also differ in the experimental setting (Table 4). More concretely, each method accepts as input a different set of genes (a.k.a. Seed genes) and a scaffolding network and requires the definition of criteria for setting thresholds or prioritization rules. For instance, the method based on clustering (referred to as MONET) starts with a partition of the scaffold network into modules (note that different clustering methods could be applied to partition the network) and then requires the definition of criteria on how to select the relevant modules, which frequently require projecting a set of seed genes into the clusters to identify those clusters that contain the genes. Some methods assume closeness in the network, while others are more flexible in this regard and do not assume that drug target genes and disease genes have to be close in the network (Ruiz et al., 2021). Thus, the results of applying each method are conditioned by the selection of input data, experimental design, and the threshold and criteria for prioritization of candidate networks and genes.

**TABLE 4:** Summary of the methods used in this study.

Clustering (MONET K1)	GUILD	iPath	MSI
• Provides a partition of the scaffold network	• Provides a ranked list of genes	• Requires selecting a list of seed genes	• Similarity of drug and disease profile
• Requires setting a criteria for selecting relevant clusters	• Requires setting a threshold to prioritize genes based on scores	• The output is a subnetwork that connects most of the genes in the seed list	• TI allows identifying relevant nodes (genes & BP)
• Mapping or enrichment analysis can be used to select clusters	• Genes can be mapped to the scaffold network to visualize how they are connected to seed and linker genes	• Can provide candidate genes, but the number is limited due to small-sized network obtained	• A subnetwork can be obtained by projecting nodes into the network
• The clusters can provide candidate genes (e.g., genes that belong to the same cluster as the seed genes; guilt-by-association principle)	• Requires selecting a list of seed genes		• Requires setting a threshold to prioritize nodes based on TI values
	• Can provide candidate genes (by selecting the appropriate threshold)		• Can provide candidate genes
			• The method has predictive capacity if trained against a benchmark of drug-disease associations

The main features of each of the methods are briefly presented. Note that the predictive capacity of the MSI was not evaluated in the current study.

The purpose of this study was not to provide insights into the potential mechanisms of liver toxicity due to VPA treatment but to illustrate the underlying assumptions, advantages, and limitations of a selection of network-based approaches. There are plenty of network-based approaches and tools available, we selected 4 of them that differ in their principles and results for illustrative purposes. Moreover, most of them require consideration of the experimental design and the definition of thresholds and filtering strategies that are more relevant to the problem under study. Since there are no clear guidelines on how the experimental setting, selection of input data, and filtering criteria should be set, the user must consider all these aspects carefully before designing an experiment and bear in mind these aspects during the interpretation of the results.

The biological interpretation of a set of genes recovered by network approaches represents a major challenge (Reimand et al., 2019) and is usually facilitated by enrichment analysis using a variety of gene annotations that provide insights on molecular functions, biological processes, pathways, regulation by transcription factors, and miRNAs, among others aspects of biology. There are many enrichment analysis tools that allow functional interpretation of gene lists, such as g:Profiler, a tool that maps genes to known functional information sources and detects statistically significantly enriched terms with the Fisher test (Raudvere et al., 2019), or others like TopGO (Alexa et al., 2006), which integrate two algorithms that use the hierarchical structure of Gene Ontology (GO) and the gene set data to obtain confident GO terms associated significantly, thus increasing the explanatory power of gene enrichment analysis. However, the enrichment analysis detects redundant GO terms, obtaining unintelligible lists which hampers biological interpretation (Reimand et al., 2019). Semantic similarity approaches to reduce those redundancies have been implemented in tools such as Revigo (Supek et al., 2011). To overcome this challenge and support the reproducibility of the analysis, we applied a systematic approach to analyze the results of each method, compare them with each other, and obtain a consensus among all methodologies.

Our analysis shows that the results of applying the different methods on a case study used as a benchmark differs in terms of the number of genes recovered and the biology these genes represent. More importantly, they differ in how well they capture the genes and biological processes of the benchmark case study. The MSI and GUILD methods showed better performance in this regard, while iPath proved to be more limited in recovering the genes and biological processes of the benchmark. Moreover, some methods, such as MONET and MSI, are more suitable to provide candidate genes and biological processes than other methods and are therefore more interesting for exploring alternative mechanistic hypotheses for a drug-disease association. This is an important feature if these methods are aimed to be used to propose the mode of action of compounds. The main features of the methods are summarized in Table 4. We also observe that each method recovers genes and biological processes that differ among them, suggesting that combining the results could provide a more complete picture of the processes underlying the drug’s effect on the disease phenotype. In addition, we present a multiscale network containing candidate genes and biological processes obtained by combining the results of the different methodologies.

By providing a detailed and systematic analysis of the outcomes of the different network-based tools, we aim at supporting users in making informed decisions on the choice of the most suitable method in the context of systems toxicology.

## Data Availability

The original contributions presented in the study are included in the article/Supplementary Material, further inquiries can be directed to the corresponding author.
